# Square-Root Sigma-Point Information Consensus Filters for Distributed Nonlinear Estimation

**DOI:** 10.3390/s17040800

**Published:** 2017-04-08

**Authors:** Guoliang Liu, Guohui Tian

**Affiliations:** School of Control Science and Engineering, Shandong University, Jinan 250061, China; g.h.tian@sdu.edu.cn

**Keywords:** target tracking, sensor network, information filter, distributed estimation

## Abstract

This paper focuses on the convergence rate and numerical characteristics of the nonlinear information consensus filter for object tracking using a distributed sensor network. To avoid the Jacobian calculation, improve the numerical characteristic and achieve more accurate estimation results for nonlinear distributed estimation, we introduce square-root extensions of derivative-free information weighted consensus filters (IWCFs), which employ square-root versions of unscented transform, Stirling’s interpolation and cubature rules to linearize nonlinear models, respectively. In addition, to improve the convergence rate, we introduce the square-root dynamic hybrid consensus filters (DHCFs), which use an estimated factor to weight the information contributions and shows a faster convergence rate when the number of consensus iterations is limited. Finally, compared to the state of the art, the simulation shows that the proposed methods can improve the estimation results in the scenario of distributed camera networks.

## 1. Introduction

Consensus based distributed estimation has attracted a lot of attention in the field, due to its outstanding performance in many applications, e.g., distributed camera networks [1], mobile sensor networks [2,3] and multi-agent systems [4,5]. Compared to the centralized estimation, the distributed estimation has many advantages, such as good scalability, low computation cost and robustness to the sensor failure [6]. In many applications, sensor nodes in the distributed network may have multiple measurements of the target state. It is important to fuse all the measurement information from sensor nodes to achieve a robust estimation result. In distributed sensor networks, there are no central fusion nodes. Instead, a fusion result or a common estimation goal can be achieved by using the novel consensus method. Consensus means reaching an agreement regarding a certain quantity of interest which depends on the state of all sensor nodes [7]. In the consensus algorithm, the node communicates to its neighbor nodes, and converges to a global mean result after a number of iterations, e.g., arithmetic mean or geometric mean [8]. Due to the limited bandwidth of the real network, only a limited number of iterations can be applied, so that the true convergence may not be always reached [8]. Therefore, the convergence rate of the consensus algorithm is very important, which is the main research aspect of this note.

In order to estimate the state of the node in the consensus architecture, the Kalman filter or information filter and their extensions are often employed [2,8,9,10,11,12,13]. In [9], a Kalman filter is used to work with the consensus algorithm, which is called Kalman consensus filter (KCF). The original KCF algorithm works well when all nodes can observe the target, but has decreased performance when the sensor node becomes naive, e.g., the node has limited observability [8]. As an alternative, the information filter is introduced to replace the Kalman filter, which is the information consensus filter (ICF) [2,10]. The information filter uses information matrix and information vector instead of the moments (mean and covariance) used in the Kalman filter to represent the Gaussian distribution. In this way, the information filter has advantages to handle sensor fusion tasks and unknown prior covariance conditions [14]. However, the ICF as well as the KCF did not address the naivety problem and the redundancy problem. The redundancy problem is caused by the iterative information exchange in consensus methods, which correlates the node estimation and delays the convergence to the optimal result [12].

To overcome the naivety and redundancy problem, an information weighted consensus filter (IWCF) was proposed in [1,15]. The IWCF solves the naivety and redundancy problem by giving less weight to the prior information when the new information contribution is fused, since the redundancy information is present only in the prior information. In addition, the IWCF can converge to the centralized solution when the number of the consensus iterations to the infinity by setting the weight as 1/N, where *N* is the number of sensor nodes. However, the sensor nodes can be overweighted in the IWCF if only a limited number of consensus iterations is performed, and the consistence of the local filter can be destroyed [11]. As an alternative weighting scheme, the inverse of an estimated factor S/N can be used to weight the new information contributions, where *S* means the number of valid sensor nodes that can observe the target currently. This choice has the desirable property of preserving the consistence of local filters and the novel information is never overestimated. Since the estimated value S/N is changing all the time due to the limited filed of view (FOV) of the sensor, we call this new filter a dynamic hybrid consensus filter (DHCF).

To handle the nonlinear system, the extended information weighted consensus filter (EIWCF) was introduced in [12], where the extended information filter is used to handle the nonlinearity. However, the IWCF and the EIWCF use a fixed consensus rate in the consensus algorithm, which is not optimal for achieving a fast convergence rate. As an alternative, the Metropolis weight and Maximum-degree weight have been proposed to work with the consensus approaches [2,11,16,17]. The Metropolis weight can achieve a faster convergence rate than the Maximum-degree weight proved in [16], which means it can achieve more accurate results when a limited number of consensus iterations is performed. Therefore, it is interesting to see how well the IWCF and the EIWCF work with the Metropolis weight. In addition, the EIWCF employs the first order of Taylor series to linearize the nonlinear models, which has low accuracy compared to other modern optimized linearization techniques, i.e., Stirling’s interpolation [18,19], unscented transform [20,21], spherical cubature rules [22,23] and their square-root extensions [14,24,25,26]. These optimized linearization methods can be called sigma-point filters since they use a number of sampled sigma-points to approximate the distribution of the state variables, and then propagate these sigma-points through the nonlinear functions to get predicted state and observations. The posterior statistics are then calculated by a weighted summation of all sigma-points. The main difference between these sigma-point filters is how to set the weights in the summation. Furthermore, these weights can affect the positive definite property of the covariance, which can make the filter numerically unstable. For instance, the parameter used in classical unscented transform can lead to the negative weights and further destroy the positive definite property of the covariance as shown in [14,27,28]. However, the cubature rules as a special kind of unscented transform is more stable by setting the parameter α=1, β=0, κ=0 [27]. On the other hand, Stirling’s interpolation also shows improved numerical performance compared to the classical unscented transform by making the weights positive [14]. Recently, a square-root cubature information weighted consensus filter (SRCIWCF) based on cubature rules has been developed for distributed object tracking, which shows that the SRCIWCF is more numerically accurate and stable than the EIWCF [13,29]. Motivated by the development of the nonlinear IWCF and its square-root extension, we developed a class of square-root sigma-point information consensus filters here, which employ Stirling’s interpolation, unscented transform and cubature rules for linearization in the framework of IWCF and DHCF, respectively. The comprehensive performance comparison between the state-of-the-art and proposed square-root sigma-point information consensus filters is also demonstrated using a simulated camera network.

The structure of this note is the following: first, the nonlinear information weighted consensus filters are introduced in Section 2, which are based on the first order of Taylor expansion, Stirling’s interpolation, unscented transform and cubature rules, respectively. Second, the nonlinear dynamic hybrid consensus filters are proposed in Section 3, which use an alternative consensus weighting scheme and can keep the consistency of local filters. Finally, a sparse of camera network is simulated to illustrate the performance of proposed square-root sigma-point information consensus methods in Section 4.

## 2. Nonlinear Information Weighted Consensus Filters

In the following sections, we consider that the sensor network has *N* nodes, which construct an undirected graph G=(C,E) where C={1,2,3,⋯,N} denotes vertex set and E⊂{{i,j}|i,j⊂C} means the edge set. The neighbor nodes of the $i_{th}$ node can be defined as $N_{i}=\left\{j\in C|\left\{i,j\right\}\in E\right\}$, which has $N_{i}$ nodes.

**Algorithm 1** Extended Information Weighted Consensus FilterInitialization: Consensus rate ϵ, number of consensus iteration *L*, process noise *Q* and measurement noise *R*.For k=1,⋯,∞:
Prediction for the next time step:
(1)$${\hat{x}}_{i,k}=f\left(x_{i,k-1}\right),$$
(2)$${\hat{Y}}_{i,k}={\left(J_{k}Y_{i,k-1}^{-1}J_{k}^{T}+W_{k}Q_{k}W_{k}^{T}\right)}^{-1},$$
(3)${\hat{y}}_{i,k}={\hat{Y}}_{i,k}{\hat{x}}_{i,k}.$Compute consensus proposals
(4)$$v_{i,k}^{0}=\frac{1}{N}{\hat{y}}_{i,k}+\phi_{i,k},$$
(5)$$V_{i,k}^{0}=\frac{1}{N}{\hat{Y}}_{i,k}+\Phi_{i,k}.$$Perform consensus on $v_{i,k}^{0}$ and $V_{i,k}^{0}$ **for** l=1 to *L* **do**
   (a)Send $v_{i,k}^{l-1}$ and $V_{i,k}^{l-1}$ to all neighbors $j\in N_{i}$   (b)Receive $v_{j,k}^{l-1}$ and $V_{j,k}^{l-1}$ from all neighbors $j\in N_{i}$   (c)Update consensus terms:
(6)$$v_{i,k}^{l}=v_{i,k}^{l-1}+\epsilon \sum_{j\in N_{i}}\left(v_{j,k}^{l-1}-v_{i,k}^{l-1}\right),$$
(7)$$V_{i,k}^{l}=V_{i,k}^{l-1}+\epsilon \sum_{j\in N_{i}}\left(V_{j,k}^{l-1}-V_{i,k}^{l-1}\right).$$
** end for**Compute the posterior at *k* time step
(8)$$y_{i,k}=Nv_{i,k}^{L},$$
(9)$$Y_{i,k}=NV_{i,k}^{L},$$
(10)$$x_{i,k}=Y_{i,k}^{-1}y_{i,k}.$$


### 2.1. Extended Information Weighted Consensus Filter

For the nonlinear system, the motion model of the target and the measurement model of the sensor can be described as

(11) $$x_{k}=f\left(x_{k-1},q_{k}\right),$$

(12) $$z_{i,k}=h_{i}\left(x_{k},v_{i,k}\right),$$

where *f* and $h_{i}$ are the state transition function and measurement function of the $i_{th}$ sensor node, respectively, and $q_{k}$ and $v_{i,k}$ are zero mean white Gaussian noises with covariance matrix $Q_{k}$ and $R_{i,k},$ respectively. In the case of the nonlinear function *f* and/or $h_{i}$, the extended information filter (EIF) can be used for the linearization. According to [12], the EIF based consensus algorithm EIWCF can be summarized as Algorithm 1. In the prediction step, the $J_{k}$ and $W_{k}$ in (2) are Jacobians of function *f* with respect to $x_{k}$ and $w_{k}$, respectively. To calculate the consensus quantity $v_{i,k}^{0}$ and $V_{i,k}^{0}$ in (4) and (5), the information contributions are derived as

(13) $$\phi_{i,k}=J_{h,k}^{T}R^{-1}\left(z_{i,k}-h_{i}\left({\hat{x}}_{i,k}\right)+J_{h,k}{\hat{x}}_{i,k}\right),$$

(14) $$\Phi_{i,k}=J_{h,k}^{T}R^{-1}J_{h,k},$$

where $J_{h,k}$ is Jacobian of function $h_{i}$ with respect to $x_{k}$.

The iterations of consensus are performed in a loop through (6) and (7), where ϵ is the consensus weight which determines the convergence rate of the algorithm. Normally, the ϵ is between 0 and $1/\Delta_{max},$ where $\Delta_{max}$ is the maximum degree of the graph *G*. The original IWCF algorithm in [8] uses a deterministic value $\epsilon =0.65/\Delta_{max}$, which is not optimal for convergence as we show in the following sections. In addition, the fixed ϵ used here requires the knowledge of the global maximum degree of the graph, which is not robust for handling the network topology changing problem. As suggested in [16], the Metropolis weights can offer a faster convergence rate without the knowledge the number of sensor nodes *N*, which is defined as

(15) $$\epsilon_{i,j,k}=\left\{\begin{array}{cc} \frac{1}{1+max\{d_{i,k},d_{j,k}\}} & ifj\in N_{i},\\ 1-\sum_{j\in N_{i}}\epsilon_{i,j,k} & ifi=j,\\ 0 & otherwise, \end{array}\right.$$

where $d_{i,k}$ and $d_{j,k}$ are the degrees of the node *i* and node *j*, respectively. The Metropolis weight only needs to know the local degree of the neighbor nodes, whereas the fixed consensus weight used in the original EIWCF algorithm requires the global knowledge of the maximum degree of the graph. Therefore, the Metropolis weight is more robust to handle the network topology changing problem. By using Metropolis weight (15) in the consensus steps (6) and (7), the new version of EIWCF algorithm can be derived which is called EIWCFM in the following part of this note.

The advantage of the EIWCF and EIWCFM would guarantee convergence to the optimal centralized estimation when the number of consensus iterations L→∞. However, due to the limited communication resources, the number of consensus iterations is limited. Therefore, it is important for the EIWCF and EIWCFM to have a faster convergence rate when a finite number of consensus iterations is performed.

### 2.2. Square-Root Central Difference Information Weighted Consensus Filter

**Algorithm 2** Square-Root Central Difference Information Weighted Consensus Filter (SRCDIWCF)
Initialization: Number of consensus iteration *L*, process noise $Q=S_{q}S_{q}^{T}$ and measurement noise $R=S_{r}S_{r}^{T}$.For k=1,⋯,∞:
Prediction for the next time step:
(16)$${\hat{x}}_{i,k}=\sum_{\tau =0}^{2m}w_{\tau }^{x}X_{i,\tau ,k|k-1}^{x},$$
(17)$${\hat{S}}_{i,k}^{x}=qr\left\{\left[A;\;B\right]\right\},$$
(18)$${\hat{S}}_{i,k}^{y}=qr\left\{{\left({\hat{S}}_{i,k}^{x}\right)}^{-1}\;I\right\},$$
(19)${\hat{y}}_{i,k}={\left({\hat{S}}_{i,k}^{x}\right)}^{-T}{\left({\hat{S}}_{i,k}^{x}\right)}^{-1}{\hat{x}}_{i,k}.$Compute consensus proposals
(20)$$v_{i,k}^{0}=\frac{1}{N}{\hat{y}}_{i,k}+\phi_{i,k},$$
(21)$$V_{i,k}^{0}=qr\left\{\left[\frac{1}{\sqrt{N}}{\hat{S}}_{i,k};\Phi_{i,k}\right]\right\}.$$Perform consensus on $v_{i,k}^{0}$ and $V_{i,k}^{0}$ **for** l=1 to *L* **do**
   (a)Send $v_{i,k}^{l-1}$ and $V_{i,k}^{l-1}$ to all neighbors $j\in N_{i}$   (b)Receive $v_{j,k}^{l-1}$ and $V_{j,k}^{l-1}$ from all neighbors $j\in N_{i}$   (c)Update consensus terms:
(22)$$v_{i,k}^{l}=v_{i,k}^{l-1}+\epsilon \sum_{j\in N_{i}}\left(v_{j,k}^{l-1}-v_{i,k}^{l-1}\right),$$
(23)$V_{i,k}^{l}=qr\left\{\left[\sqrt{1-\epsilon N_{i}}V_{i,k}^{l-1};\;\sqrt{\epsilon }V_{j=1,k}^{l-1};\;\cdots ;\sqrt{\epsilon }V_{j=N_{i},k}^{l-1}\right]\right\}.$ **end for**Compute the posterior at *k* time step
(24)$$y_{i,k}=Nv_{i,k}^{L},$$
(25)$$S_{i,k}^{y}=\sqrt{N}V_{i,k}^{L},$$
(26)$$x_{i,k}={\left(S_{i,k}^{y}\right)}^{-T}{\left(S_{i,k}^{y}\right)}^{-1}y_{i,k}.$$


The core of the proposed square-root central difference information weighted consensus filter (SRCDIWCF) is the Stirling’s interpolation for linearization. It first generates a number of sample sigma-points according to the current augmented state and covariance. The sampled state $X_{i,\tau ,k-1}^{x}$ together with its sampled process noise $X_{i,\tau ,k-1}^{q}$ construct the sampled augmented state $X_{i,\tau ,k-1}^{aq}={\left[X_{i,\tau ,k-1}^{x}\;X_{i,\tau ,k-1}^{q}\right]}^{T}$ generated by

(27) $$X_{i,\tau ,k-1}^{aq}=\left\{\begin{array}{cc} x_{i,k-1}^{aq}, & \tau =0,\\ x_{i,k-1}^{aq}+{\left(hS_{i,k-1}^{aq}\right)}_{\tau }, & \tau =1,\cdots ,m,\\ x_{i,k-1}^{aq}-{\left(hS_{i,k-1}^{aq}\right)}_{\tau }, & \tau =m+1,\cdots ,2m, \end{array}\right.$$

where $x_{i,k-1}^{aq}={\left[x_{i,k-1}\;{\bar{q}}_{i,k-1}\right]}^{T}$ and $S_{i,k-1}^{aq}=\sqrt{P_{i,k-1}^{aq}}=\sqrt{diag\{P_{i,k-1},Q_{i,k-1}\}}$ are the augmented state and square root of augmented covariance, τ indicates the $\tau_{th}$ column of the matrix, and *m* is the dimension of the augmented state. The parameter h≥1 is the scalar central difference step size. If the random variables obey a Gaussian distribution, the optimal value of *h* is $\sqrt{3}$ [19]. We can see that the square root calculation of the covariance in (27) requires that the covariance matrix $P_{i,k-1}^{aq}$ must be symmetric and positive definite. However, due to the errors introduced by arithmetic operations performed on finite word-length digital computers, or ill-conditioned nonlinear filtering problems, the positive definite property of the covariance can be destroyed. In the literature, the square-root representation of the covariance is preferred to handle such an issue. Therefore, we here present the SRCDIWCF summarized in Algorithm 2, which can avoid square-root operation, improve numerical accuracy, have double order precision and preserve symmetry of the covariance.

After the generation of the sigma-points in (27), we can propagate them through the nonlinear state transition function of (11), and sum them up to derive the predicted state as in (16). Therefore, no Jacobian matrix calculation is required here. The predicted square-root of covariance ${\hat{S}}_{i,k}^{x}$ can be calculated using QR decomposition as in (17), where

(28) $$A=\sqrt{w_{1}^{p_{1}}}\left(X_{i,1:m,k|k-1}^{x}-X_{i,m+1:2m,k|k-1}^{x}\right),$$

(29) $$B=\sqrt{w_{1}^{p_{2}}}\left(X_{i,1:m,k|k-1}^{x}+X_{i,m+1:2m,k|k-1}^{x}-2X_{i,0,k|k-1}^{x}\right).$$

The corresponding weights for the predicted mean and square-root of covariance are defined as

(30) $$\begin{array}{cc} w_{0}^{x}=\frac{h^{2}-m}{h^{2}},\\ w_{\tau }^{x}=\frac{1}{2h^{2}},\\ w_{\tau }^{p_{1}}=\frac{1}{4h^{2}},\\ w_{\tau }^{p_{2}}=\frac{h^{2}-1}{4h^{4}}, & \tau =1,\cdots ,2m, \end{array}$$

where we can see that weights $w_{\tau }^{p_{1}}$ and $w_{\tau }^{p_{1}}$ for the covariance updating are all non-negative values since h≥1, which is an important property of the proposed SRCDIWCF, since it can protect the positive property of the covariance matrix and further improve the numerical characteristics of the proposed method [14]. Because we are interested in the information form representation, the predicted information vector and information matrix can be computed from predicted mean and covariance as shown in (19) and (18), respectively.

The second step is to compute the consensus quantities $v_{i,k}^{0}$ and $v_{i,k}^{0}$ defined as (20) and (21), where $\phi_{i,k}$ and $\Phi_{i,k}$ are information contributions calculated as

(31) $$\Phi_{i,k}={\hat{S}}_{i,k}^{y}{\left({\hat{S}}_{i,k}^{y}\right)}^{T}{\hat{P}}_{i,xz}S_{r,i,k}^{-T},$$

(32) $$\phi_{i,k}=\Phi_{i,k}{\left(S_{r,i,k}\right)}^{-1}\left(z_{i,k}-{\hat{z}}_{i,k}+{\hat{P}}_{i,xz}^{T}{\hat{y}}_{i,k}\right),$$

where $z_{i,k}$ and ${\hat{z}}_{i,k}$ are real and predicted sensor measurements of the target object, respectively. The predicted ${\hat{z}}_{i,k}$ can be calculated using Stirling’s interpolation according to the predicted state of the target as

(33) $${\hat{z}}_{i,k}=\sum_{\tau =0}^{2m}w_{\tau }^{x}Z_{i,\tau ,k|k-1},$$

(34) $$Z_{i,\tau ,k|k-1}=h\left(X_{i,\tau ,k|k-1}\right),$$

(35) $$X_{i,\tau ,k|k-1}=\left\{\begin{array}{cc} {\hat{x}}_{i,k}, & \tau =0,\\ {\hat{x}}_{i,k}+{\left(h{\hat{S}}_{i,k}^{x}\right)}_{\tau }, & \tau =1,\cdots ,m,\\ {\hat{x}}_{i,k}-{\left(h{\hat{S}}_{i,k}^{x}\right)}_{\tau }, & \tau =m+1,\cdots ,2m, \end{array}\right.$$

where $X_{i,\tau ,k|k-1}$ is the generated sigma point according to the predicted state and covariance, and $Z_{i,\tau ,k|k-1}$ is the predicted sigma point of the measurement. The cross-covariance ${\hat{P}}_{i,xz}$ between the state and the measurement calculated as

(36) $${\hat{P}}_{i,xz}=\sqrt{w_{1}^{p_{1}}}{\hat{S}}_{i,k}^{x}{\left(Z_{i,1:m}-Z_{i,m+1:2m}\right)}^{T}.$$

From (20) and (21), we can see that both the prior information vector ${\hat{y}}_{i,k}$ and prior information matrix ${\hat{Y}}_{i,k}$ are weighted by 1/N. The reason to do this weighting is to remove the information redundancy during information sharing between sensor nodes.

The third step is to perform consensus iteratively. The sensor node exchanges the consensus quantities $v_{i,k}^{l}$ and $V_{i,k}^{l}$ with neighbor nodes for *L* steps, and then update its local estimation using (22) and (23), where $\epsilon \in (0,1/\Delta_{max})$ is defined as the same constant parameter as the EIWCF presented in [12]. If the Metropolis weight is used for ϵ instead of the fixed one, the SRCDIWCF is called SRCDIWCFM for distinguishment. Therefore, the basic idea of the consensus is to get the weighted summation of information quantities, so the information from neighbor nodes can be fused. To output the final results, the fourth step is to derive the estimated information vector $y_{i,k}$, information matrix $S_{i,k}^{y}$ and state $x_{i,k}$ from the final information quantities using (24)–(26).

### 2.3. Square-Root Unscented Information Weighted Consensus Filter

Here, we present the unscented transform based square-root unscented information weighted consensus filter (SRUIWCF). The main difference between the SRUIWCF and SRCDIWCF is the weighting scheme for the sigma points to calculate the predicted mean, covariance and information contributions. For the SRUIWCF, the sigma-points are generated by

(37) $$X_{i,\tau ,k-1}^{aq}=\left\{\begin{array}{cc} x_{i,k-1}^{aq}, & \tau =0,\\ x_{i,k-1}^{aq}+{\left(\gamma S_{i,k-1}^{aq}\right)}_{\tau }, & \tau =1,\cdots ,m,\\ x_{i,k-1}^{aq}-{\left(\gamma S_{i,k-1}^{aq}\right)}_{\tau }, & \tau =m+1,\cdots ,2m, \end{array}\right.$$

where $\gamma =\sqrt{\left(\lambda +m\right)}$ is the composite scaling parameter, *m* is the dimension of the state, and $\lambda =\alpha^{2}\left(m+\kappa \right)-m$. α and κ are scaling parameters that determine how far the sigma points spread from the mean value. The predicted mean and the square-root of covariance can be derived by

(38) $${\hat{x}}_{i,k}=\sum_{\tau =0}^{2m}w_{\tau }^{x}X_{i,\tau ,k|k-1}^{x},$$

(39) $${\hat{S}}_{i,k}^{x}=cholupdate\left\{C,D,sign\left\{w_{0}^{p}\right\}\right\},$$

(40) $$C=qr\{\sqrt{w_{1}^{p}}\left(X_{i,1:2m,k|k-1}^{x}-{\hat{x}}_{i,k}\right)\},$$

(41) $$D=\sqrt{w_{0}^{p}}\left(X_{i,0,k|k-1}^{x}-{\hat{x}}_{i,k}\right),$$

where the weights defined by

(42) $$\begin{array}{cc} w_{0}^{x}=\frac{\lambda }{m+\lambda },\\ w_{0}^{p}=\frac{\lambda }{m+\lambda }+\left(1-\alpha^{2}+\beta \right),\\ w_{\tau }^{x}=w_{\tau }^{p}=\frac{1}{2(m+\lambda )}, & \tau =1,\cdots ,2m. \end{array}$$

Since the weight $w_{0}^{p}$ might be negative, we need an additional *cholupdate* to update the *Cholesky factor* ${\hat{S}}_{i,k}^{x}$ in (39), whereas the SRCDIWCF does not need this step since all weights used for the covariance update are positive. The negative update might destroy the positive definite property of the Cholesky factor, such that the SRCDIWCF is preferable to the SRUIWCF concerning the numerical stability.

For calculating information contributions, the predicted measurement $Z_{i,\tau ,k|k-1}$ and the cross-covariance ${\hat{P}}_{i,xz}$ are given by

(43) $${\hat{z}}_{i,k}=\sum_{\tau =0}^{2m}w_{\tau }^{x}Z_{i,\tau ,k|k-1},$$

(44) $${\hat{P}}_{i,xz}=\sum_{\tau =0}^{2m}w_{\tau }^{p}\left(X_{i,\tau ,k|k-1}-{\hat{x}}_{i,k}\right){\left(Z_{i,\tau ,k|k-1}-{\hat{z}}_{i,k}\right)}^{T}.$$

The consensus quantities and iterations steps of the SRUIWCF are same as the SRCDIWCF.

### 2.4. Square-Root Cubature Information Weighted Consensus Filter

Basically, the cubature rule is a special case of the unscented transform defined by

(45) $$X_{i,\tau ,k-1}^{aq}=\left\{\begin{array}{cc} x_{i,k-1}^{aq}+{\left(\sqrt{m}S_{i,k-1}^{aq}\right)}_{\tau }, & \tau =1,\cdots ,m,\\ x_{i,k-1}^{aq}-{\left(\sqrt{m}S_{i,k-1}^{aq}\right)}_{\tau }, & \tau =m+1,\cdots ,2m, \end{array}\right.$$

and the weights used for calculating posterior mean and covariance given by

(46) $$\begin{array}{cc} w_{\tau }^{x}=w_{\tau }^{p}=\frac{1}{2m}, & \tau =1,\cdots ,2m. \end{array}$$

By setting the parameter of the unscented transform as α=1, β=0 and κ=0, the unscented transform becomes the cubature rule [27,28]. Therefore, the square-root cubature information weighted consensus filter (SRCIWCF) can be derived from SRUIWCF by using this specific parameter. Since the weights used in SRCIWCF are positive, the SRCIWCF is more numerically stable than the SRUIWCF.

## 3. Nonlinear Dynamic Hybrid Consensus Filters

The IWCFs can converge to the centralized solution when the number of consensus *L* is infinity. However, for the real-time requirements of the real applications, *L* is usually small. In such a case, the weight 1/N used in IWCFs can overweight the prior information for some sensor nodes, which can affect the convergence rate of the algorithm. Here, we present an alternative way to weight the information, in order to ensure that the sensor node never get overweighted, and has a faster convergence rate if a small *L* is used.

**Algorithm 3** Square-Root Central Difference Dynamic Hybrid Consensus Filter (SRCDDHCF)Initialization: Number of consensus iteration *L*, process noise $Q=S_{q}S_{q}^{T}$ and measurement noise $R=S_{r}S_{r}^{T}$.For k=1,⋯,∞:
Prediction for the next time step:
(47)$${\hat{x}}_{i,k}=\sum_{\tau =0}^{2m}w_{\tau }^{x}X_{i,\tau ,k|k-1}^{x},$$
(48)$${\hat{S}}_{i,k}^{x}=qr\left\{\left[A;\;B\right]\right\},$$
(49)$${\hat{S}}_{i,k}^{y}=qr\left\{{\left({\hat{S}}_{i,k}^{x}\right)}^{-1}\;I\right\},$$
(50)${\hat{y}}_{i,k}={\left({\hat{S}}_{i,k}^{x}\right)}^{-T}{\left({\hat{S}}_{i,k}^{x}\right)}^{-1}{\hat{x}}_{i,k}.$Compute consensus proposals **if** i∈S **then**
(51)$$\Phi_{i,k}={\hat{S}}_{i,k}^{y}{\left({\hat{S}}_{i,k}^{y}\right)}^{T}{\hat{P}}_{i,xz}S_{r,i,k}^{-T},$$
(52)$$\phi_{i,k}=\Phi_{i,k}{\left(S_{r,i,k}\right)}^{-1}\left(z_{i,k}-{\hat{z}}_{i,k}+{\hat{P}}_{i,xz}^{T}{\hat{y}}_{i,k}\right),$$
(53)$$b_{i,k}=1,$$
 **else**
(54)$$\Phi_{i,k}=0,\;\phi_{i,k}=0,\;b_{i,k}=0.$$
 **end if**Perform hybrid consensus iterations  **Initialization:** $b_{i,k}^{0}=b_{i,k}$, $\left({\hat{y}}_{i,k}^{0}={\hat{y}}_{i,k},{\hat{S}}_{i,k}^{0}={\hat{S}}_{i,k}^{y}\right)$ and $\left(\phi_{i,k}^{0}=\phi_{i,k},\Phi_{i,k}^{0}=\Phi_{i,k}\right)$  **for** l=1 to *L* **do**
  (a)Send $b_{i,k}^{l-1}$, $\left({\hat{y}}_{i,k}^{l-1},{\hat{S}}_{i,k}^{l-1}\right)$ and $\left(\phi_{i,k}^{l-1},\Phi_{i,k}^{l-1}\right)$ to all neighbors $j\in N_{i}$  (b)Receive $b_{i,k}^{l-1}$, $\left({\hat{y}}_{i,k}^{l-1},{\hat{S}}_{i,k}^{l-1}\right)$ and $\left(\phi_{i,k}^{l-1},\Phi_{i,k}^{l-1}\right)$ from all neighbors $j\in N_{i}$  (c)Update consensus terms:
(55)$$y_{i,k}^{l}=\sum_{j\in N_{i}}\epsilon_{i,j,k}y_{j,k}^{l-1},$$
(56)$$S_{i,k}^{l}=qr\left\{\left[\sqrt{\epsilon_{i,j,k}}S_{j=1,k}^{l-1};\;\cdots ;\sqrt{\epsilon_{i,j,k}}S_{j=N_{i},k}^{l-1}\right]\right\},$$
(57)$$\phi_{i,k}^{l}=\sum_{j\in N_{i}}\epsilon_{i,j,k}\phi_{j,k}^{l-1},$$
(58)$$\Phi_{i,k}^{l}=qr\left\{\left[\sqrt{\epsilon_{i,j,k}}\Phi_{j=1,k}^{l-1};\;\cdots ;\sqrt{\epsilon_{i,j,k}}\Phi_{j=N_{i},k}^{l-1}\right]\right\},$$
(59)$$b_{i,k}^{l}=\sum_{j\in N_{i}}\epsilon_{i,j,k}b_{j,k}^{l-1}.$$
  **end for**Compute the posterior at *k* time step
(60)$$\omega_{i,k}^{L}=\left\{\begin{array}{cc} 1/b_{i,k}^{L} & if\;b_{i,k}^{L}\neq 0,\\ 1 & otherwise, \end{array}\right.$$
(61)$$y_{i,k}={\hat{y}}_{i,k}^{L}+\omega_{i,k}^{L}\phi_{i,k}^{L},$$
(62)$$S_{i,k}^{y}=qr\left\{\left[S_{i,k}^{L};\sqrt{\omega_{i,k}^{L}}\Phi_{i,k}^{L}\right]\right\},$$
(63)$$x_{i,k}={\left(S_{i,k}^{y}\right)}^{-T}{\left(S_{i,k}^{y}\right)}^{-1}y_{i,k}.$$


To keep the consistence of local filters, the inverse of an estimated factor S/N can be used for weighting information contributions derived by

(64) $$\omega_{i,k}^{l}=\left\{\begin{array}{cc} 1/b_{i,k}^{l} & if\;b_{i,k}^{l}\neq 0,\\ 1 & otherwise, \end{array}\right.$$

where $b_{i,k}^{l}$ is an estimation of the fraction S/N at $l_{th}$ iterative step via the consensus algorithm

(65) $$b_{i,k}^{l}=\sum_{j\in N_{i}}\epsilon_{i,j,k}b_{j,k}^{l-1}.$$

The initialization of $b_{i,k}^{0}$ is set as 1 if the $i_{th}$ node can observe the target, i.e., i∈S, where S means the set of these effective nodes, whereas it is set as 0 for other nodes as shown in (54). Here, the consensus weight $\epsilon_{i,j,k}$ employs the Metropolis weight. In such a way, the quantity $\omega_{i,k}^{L}\epsilon_{i,j,k}\leq 1$ for any pair of (i,j) since $b_{i,k}^{L}=\sum_{j\in N_{i}}\epsilon_{i,j,k}$. Therefore, the information contributions will not get overweighted and the consistence of the local filter has been kept [11]. In addition, compared to the IWCFs, no prior knowledge of network connections is required here. To summarize, based on this new weighting schemes, the square-root central difference dynamic hybrid consensus filter (SRCDHCF) can be derived as in Algorithm 3. In a similar way, the square-root unscented dynamic hybrid consensus filter (SRUDHCF) and the cubature dynamic hybrid consensus filter (SRCDHCF) can be further developed. However, the DHCFs require more consensus quantities to be shared with other neighbor nodes than IWCFs, i.e., prior information $\left({\hat{y}}_{i,k}^{l-1},{\hat{S}}_{i,k}^{l-1}\right)$ , information contributions $\left(\phi_{i,k}^{l-1},\Phi_{i,k}^{l-1}\right)$ and $b_{i,k}^{l-1}$, which means the algorithms of DHCFs require more data bandwidth.

## 4. Simulation

To show the performance of the proposed square-root sigma-point information consensus filters, a sparse network is simulated using nine cameras ($c_{1}$ to $c_{9}$) as shown in [12], which has a maximum connection degree $\Delta_{max}=2$. Each camera has a fixed field of view (FOV), i.e., 200×200. When the target moves, some of the camera nodes may lose the measurement information. Therefore, the estimated value S/N for the algorithm DHCFs is time varying,

In this simulation, the state transition model and measurement model of the object are nonlinear functions. The state of the target is defined as $x_{k}={\left[x_{k},y_{k},v_{x,k},v_{y,k},\delta_{k}\right]}^{T}$, and the motion model of the target is given as

(66) $$x_{k+1}=\left[\begin{array}{c} x_{k}+v_{x,k}\delta_{k}+a_{x}\delta_{k}^{2}/2\\ y_{k}+v_{y,k}\delta_{k}+a_{y}\delta_{k}^{2}/2\\ v_{x,k}+a_{x}\delta_{k}\\ v_{y,k}+a_{y}\delta_{k}\\ \delta_{k}+e \end{array}\right],$$

where $\left(x_{k},y_{k}\right)$, $\left(v_{x,k},v_{y,k}\right)$ and $\left(a_{x},a_{y}\right)$ are the position, velocity and acceleration of the target, respectively. The acceleration is modeled as Gaussian noise. $\delta_{k}$ is the time step between two consecutive measurements. The synchronization error among cameras is also considered as a Gaussian variable *e*. The vector $w={\left[a_{x},a_{y},e\right]}^{T}$ is considered as the Gaussian noise vector with zero mean and covariance Q=diag{1,1,0.01}. We consider a nonlinear measurement model of the camera node *i* as

(67) $$z_{i,k}=\left[\begin{array}{c} u_{i,k}\\ v_{i,k} \end{array}\right]=\left[\begin{array}{c} \frac{H_{11}x_{k}+H_{12}y_{k}+H_{13}}{H_{31}x_{k}+H_{32}y_{k}+H_{33}}\\ \frac{H_{21}x_{k}+H_{22}y_{k}+H_{23}}{H_{31}x_{k}+H_{32}y_{k}+H_{33}} \end{array}\right]+v_{i,k},$$

where $\left(u_{i,k},v_{i,k}\right)$ is the pixel coordinates of the target in the image, and $\left(H_{11},\cdots ,H_{33}\right)$ are elements of the homography matrix defined as

(68) $$H=\left[\begin{array}{ccc} 397.2508 & 95.2020 & 287280\\ 51.7437 & 396.9189 & 139100\\ 0.0927 & 0.1118 & 605.2481 \end{array}\right],$$

which are taken from one of the cameras of the APIDIS dataset [12].

### 4.1. Normal Measurement

The measurement noise $v_{i,k}$ is defined as Gaussian noise with zero mean and covariance R=diag{15,15}. The initial covariance matrix of the state for each camera node is set to be a diagonal matrix P=diag{0.1,0.1,0.1,0.1,0.0001}.

**Figure 1:**
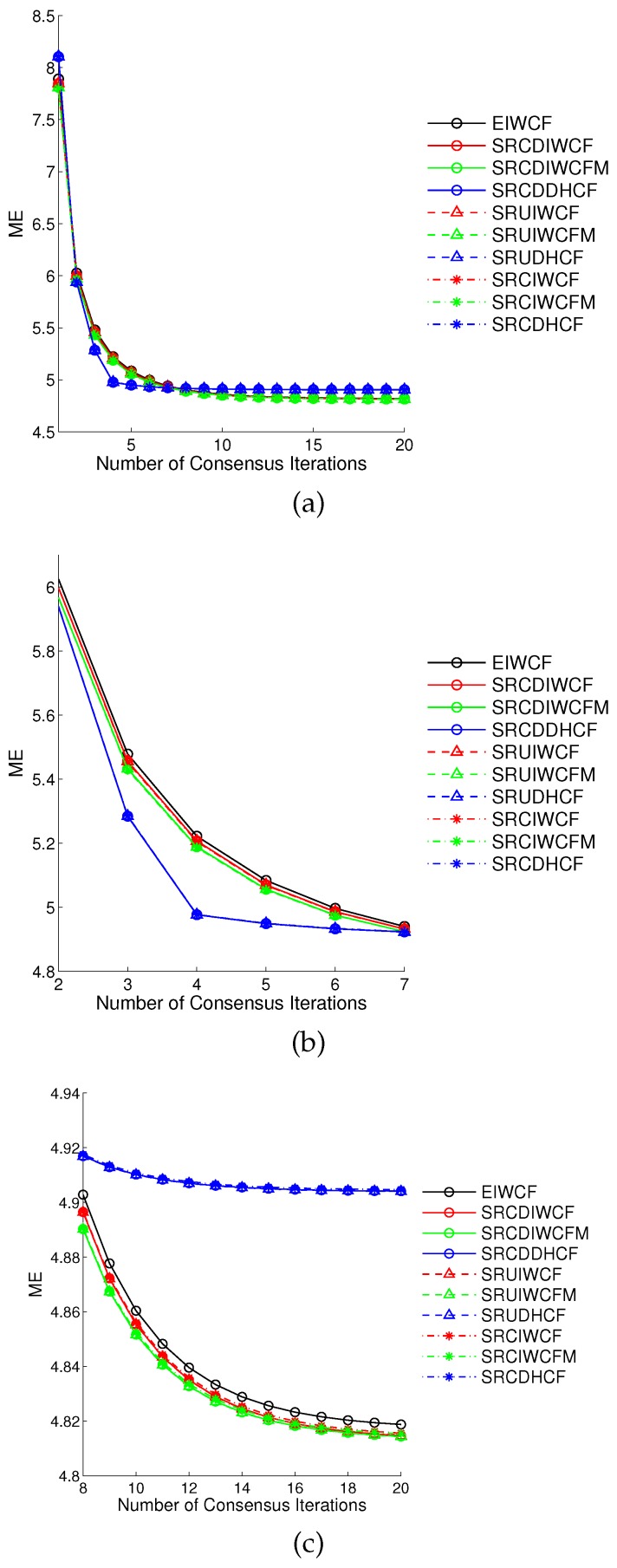
The comparison of mean errors (ME) of proposed square-root sigma-point information consensus filters and the state-of-the-art extended information weighted consensus filter (EIWCF) for 50 Monte Carlo simulations. The details of the (**a**) is shown in (**b**) for iterations 2–7 and (**c**) for iterations 8–20.

We here demonstrate the simulation for 50 Monte Carlo runs, and the result is shown in Figure 1. Figure 1a shows the overall performance comparison for the number of consensus iterations *L* from 1 to 20, whereas the Figure 1b shows the first half part of Figure 1a for *L* from 2 to 7, and the Figure 1c shows the second half part of Figure 1a for *L* from 8 to 20. By analyzing the result data, we can derive following conclusions: first, the square-root sigma-point information consensus filters outperform the EIWCF as shown in Figure 1b,c, since they can capture higher order terms of Taylor expansion. Because the dimension of the state is small, the differences between the Stirling’s interpolation, unscented transform and cubature rule based methods are minor, e.g., the SRCDDHCF, SRUDHCF and SRCDHCF are almost overlapped in Figure 1 (blue lines). Second, the DHCFs have a faster convergence rate than IWCFs, i.e., the DHCFs are close to a convergence state at the iteration L=4 as shown in Figure 1b. The reason is that IWCFs based methods can overweight some sensor nodes, which can decrease the convergence rate for smaller consensus iteration value *L*. Third, the IWCFs can achieve more accurate results than DHCFs by running more iterations, e.g., L>7. Fourth, the Metropolis weight indeed can improve the performance of IWCFs compared with the fixed one, e.g., SRCDIWCFM, SRUIWCFM and SRCIWCFM using Metropolis weight have better performance than SRCDIWCF, SRUIWCF and SRCIWCF using $0.65/\Delta_{max}$. Therefore, the Metropolis weight is more preferred for square-root sigma-point information consensus filters.

### 4.2. Ill Condition: Near Perfect Measurement

**Figure 2:**
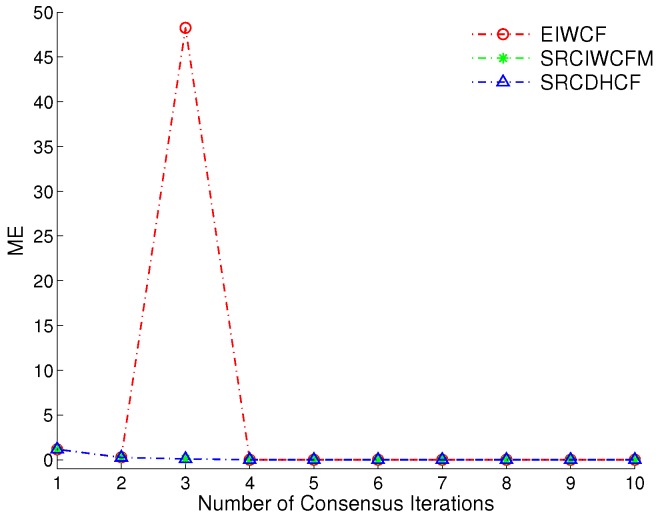
The comparison of mean errors (ME) of square-root cubature information weighted consensus filter with Metropolis weights (SRCIWCFM), square-root cubature dynamic hybrid consensus filter (SRCDHCF) and the original EIWCF for 20 Monte Carlo simulations.

The ill condition can lead the filter system to be unstable due to the accumulated round-off errors in the computational system. The square-root filters have shown their advantages with handling such problems in previous publications [13,14,25,26]. Here, we use a similar idea to compare the performance of SRCIWCFM, SRCDHCF and original EIWCF by setting the measurement covariance to be a very small value $R=diag\{2^{-52},2^{-52}\}$ for a normal 32-bit operational system. The simulations are demonstrated for 20 Monte Carlo runs with the iterations L=1,⋯,10. The experimental results are shown in Figure 2. We can see that the performance of SRCIWCFM and SRCDHCF are very close to each other, whereas the EIWCF becomes unstable when the iteration number is equal to 3. In addition, the simulation software MATLAB (R2010a, The MathWorks Inc, Natick, MA, USA) always shows the warning message “Matrix is close to singular or badly scaled” when the EIWCF algorithm runs with the near perfect measurements. In contrast, the SRCIWCFM and SRCDHCF are robust to such ill conditions and converge to a very stable result.

## 5. Conclusions

In this paper, we proposed two kinds of square-root sigma-point information consensus filters, which are based on the IWCF and DHCF methods, respectively. By comparing to the state-of-the-art method, the proposed methods can achieve more accurate estimation results than the EIWCF by using Stirling’s interpolation, unscented transform and cubature rules for linearization of nonlinear models. Furthermore, we also show that the DHCF based methods are preferred for a small number of consensus iterations, since they can achieve faster convergence rate. However, the IWCF based methods can offer more accurate results when the number of consensus iterations is large enough, especially when the redundancy information between the sensor nodes is equally distributed. Currently, the topology of sensor networks in our experiment has a fixed connection, which is a limitation if the sensors are dynamic, e.g., robots. An interesting and possible future extension of current work is using an M-matrix approach to handle such issues [30,31].
